# Targeted Endometrial Scratching: An Example of Endometrial Diagnosis Usage in Reproductive Medicine

**DOI:** 10.3389/fimmu.2020.589677

**Published:** 2020-09-30

**Authors:** Mona Rahmati, Nathalie Lédée

**Affiliations:** ^1^ London Women’s Clinic, London, United Kingdom; ^2^ MatriceLAB Innove, Pépinière Paris Santé Cochin, Hôpital Cochin, Paris, France; ^3^ Centre d’Assistance Médicale à la Procréation, Hôpital des Bluets, Paris, France

**Keywords:** embryo implantation, endometrial scratching, repeated implantation failure, endometrial receptivity, reproductive medicine

## Introduction

Over the last ten years, debates regarding the potential benefit of an endometrial scratching to enhance embryo implantation have been continuous. Despite randomised trials unable to show clear benefits, the controversy is still ongoing with the passionate defenders of empirical usage of the endometrial scratching.

The biological rationale behind this procedure is linked to the endometrial pseudo-inflammatory reaction happening in mid-luteal phase and enabling embryo implantation. An endometrial scratching is believed to enhance the local inflammation and improve embryo implantation.

The clinical studies on endometrial scratching focus on its interest on unselected groups of patients experiencing repeated unexplained embryo implantation failure, some more globally on cases of unexplained infertility, or even on good prognosis patients going through fertility treatment. Majority of reports on endometrial scratching do not use any endometrial diagnostic tool beforehand to confirm the actual need to enhance the local inflammation.

In this manuscript, we will first focus on the immune rationale linking endometrial inflammation and embryo implantation, the potential biomarkers of endometrial inflammation, as well as the local effects of an endometrial scratching. We will then illustrate the predominant clinical usage of a blind endometrial scratching in reproductive medicine, reported in most of dedicated publications. Finally, we will discuss the possible usage of a targeted endometrial scratching once the local diagnosis of an endometrial lack of inflammation is established.

Our objective is to emphasize the role of endometrial diagnosis when facing repeated failure in reproductive medicine and to draw some directions of research to study further the potential utility of a targeted endometrial scratching in selected cases.

## Immune Rationale: Endometrial Inflammation and Embryo Implantation

In the 80’s, researchers documented the association of mechanical manipulation with decidual formation in rodents (1, 2) and used this system to trigger the endometrial decidualization of foster mothers in case of embryo transfer. By analogy, they started to explore the effect of a local injury in human endometrium.

Mid-luteal phase in human endometrium is described as the window of implantation. As early as 2010, Gnainsky et al. (3) reported that a local injury specifically performed in the mid-luteal phase modified the endometrial expression occurring at the subsequent mid-luteal phase. They then documented the endometrial expression in two distinct groups within patients experiencing repeated embryo implantation failure: one experimental group with local injury and one control group without local injury.

The analysis of some cytokines of interest in embryo implantation showed higher concentrations in day-21 endometrial samples in the group with scratching when compared with the control group. The proteins reported were Growth-Regulated Oncogene-*α* (GRO-*α*), Interleukin-15 (IL-15), Macrophage Inflammatory Protein 1B (MIP-1B) and Tumor Necrosis Factor *α* (TNF-*α*).

Using flow cytometry, they observed higher amount of macrophages and dendritic cells (DC) in the day-21 endometrial samples of patients with scratching compared to day-21 samples of the control group. Additionally, this increase was observed when comparing day-21 endometrial samples of patients with scratching to earlier samples in the same group (day-8 to 10). This observation was in concordance with earlier reports showing the peak recruitment of DCs and macrophages in the human endometrium during the window of implantation (4, 5).

In 2015, the same team demonstrated that endometrial scratching upregulated the expression of pro-inflammatory cytokines involved in the recruitment of monocytes and their differentiation into macrophages and DCs (6). These cells are known to trigger the expression of pro-implantation genes in endometrial epithelium and stroma, enabling the apposition and adhesion of the blastocyst.

The role of DCs and macrophages in human endometrium at the time of implantation still needs clarification. However studies on murine models provided strong evidence of their essential intervention in implantation (7, 8). DCs and macrophages were shown to secrete an array of cytokines and enzymes allowing tissue remodelling and angiogenesis, required for the endometrial decidualization and regulation of trophoblast invasion (8–10). This differentiation of stromal cells within the decidualization process is an essential step for a successful implantation (11).

Yu Liang also explored the effect of the endometrial scratching on cytokine secretion in women with unexplained subfertility (12). This team was able to confirm the induced increase of pro-inflammatory cytokines previously reported. Likewise, they documented the scratching-induced increase of VEGF (Vascular Endothelial Growth Factor), a central growth factor involved in local angiogenesis and placentation.

The uterine Natural Killer cells (uNK) are another essential immune cell population in the human endometrium. Described as CD56^bright^ CD16^dim^, uNKs are found in the endometrium at the beginning of each menstrual cycle as small agranular cells. Within the cycle, they grow and develop numerous mediator-filled granules. They represent the majority of endometrial immune cells during the window of implantation. In contrast to the peripheral NKs (pNKs), the uNKs show no cytotoxic activity and produce high amounts of factors required for embryo implantation, including Transforming Growth Factor (TGF)-β, VEGF and Interferon (IFN)-γ (13, 14). Our team has previously shown, during mid luteal phase, a variation of levels of uNKs in some patients facing repeated implantation failure, when compared to the endometrium of fertile controls (15).

The differentiation, proliferation, activation, and survival of NKs are regulated by IL-15 (16–18). In the endometrium as well, IL-15 is the central cytokine for the uNKs recruitment and maturation enabling them to acquire their effective pro-implantation functions (17, 18). Similarly, our team has described variable levels of endometrial IL-15 in groups of patients with repeated implantation failure when compared to the controls (19).

As suggested by these studies, selected endometrial cytokines or immune cells could be considered as relevant candidates to become the biomarkers of the local immune activation or inflammation, for example the endometrial cytokine levels (MIP-1B, IL-15, TNF, VEGF) or the endometrial immune cell recruitment (DCs, macrophages, uNKs). Variations of these biomarkers would establish the initial endometrial diagnosis of the lack of local immune reaction, indicating the potential need for an endometrial scratching to promote the local inflammation and subsequently embryo implantation.

Surprisingly, only few clinical teams tried to establish an endometrial immune diagnosis prior to using the scratching. When considering randomized trials, none used immune endometrial parameters to possibly select the patients who would benefit from an endometrial scratching.

## Blind Endometrial Scratching in Clinical Practice

Within the teams reporting the blind usage of endometrial scratching, a variety of different timings have been reported. The majority seems to perform a single endometrial scratching during mid-luteal phase of the cycle preceding the actual treatment cycle. But endometrial scratching during the proliferative phase (20, 21), at the time of oocyte retrieval (22) or under contraceptive pill (23) have also been reported. Given the biological rational of an endometrial scratching as previously described, focusing on the mid-luteal phase, at the time of theoretical endometrial decidualization and peak of immune recruitment, might appear more coherent but is still not fundamentally confirmed.

The clinical frame in which a blind endometrial scratching is performed remains undefined. The scratching without pre-established endometrial diagnosis has been reported with different types of treatment: IVF treatment with fresh or frozen embryo transfers, intra uterine insemination (IUI), or treatment with donor eggs. A large number of small RCTs have been reviewed by Li et al. (24) including 25 with IVF treatment and 12 with IUI. The methodological issues were detailed with no clear benefit identified.

The inclusion criteria for such procedure remains also unclear even at clinical scale, hence with blurred outcomes. The blind endometrial scratching was attempted in patients with a history of repeated unexplained embryo implantation failure, with a history of unexplained repeated miscarriages, with global unexplained infertility and even in attempts of good prognosis. Regarding this last group, recent RCTs and meta-analysis again reported an absence of benefit, when blind endometrial scratching was attempted on patients undergoing their first embryo transfer (25), on good prognosis patients having their first cycle of IVF (26), or on patients using donor eggs (27).

## Clinical Experience With Targeted Endometrial Scratching

Despite the fundamental study of multiple endometrial biomarkers of the local immune activation, or the pseudo-inflammatory reaction in mid-luteal phase, their clinical applications remain sparse.

In legitimate first position, the uNKs, representing the majority of indispensable endometrial immune cells, were explored as potential biomarkers. Different methods were attempted to standardise their endometrial count (28) but the lone quantitative assessment doesn’t seem to be sufficient (29).

The strategy we developed was based on the evaluation of the recruitment and maturity of the uNKs in mid-luteal phase. In this view, we performed the count of CD56 positive uNKs along with the assessment of expression levels for IL-15 and its modulator Fn-14 (Fibroblast growth factor-inducible molecule) on timed endometrial biopsies obtained with a pipelle (30). This diagnostic biopsy was performed on patients with a history of unexplained repeated embryo implantation failure. The endometrial scratching was recommended only if the patient was diagnosed with a lack of uNK recruitment or maturity. The endometrial scratching would take place during the mid-luteal phase of the preceding cycle to the actual embryo transfer.

Based on our previous published cohort studies (31, 32), in a population of 1145 patients with a history of unexplained repeated embryo implantation failure, only 33.5% (384) showed a lack of uNKs recruitment or maturity in mid-luteal phase and therefore a possible indication of an endometrial scratching. In the remaining population, 19.4% (223) had a balanced immune endometrial profile and 47% (539) showed an endometrial hyper inflammation, where an endometrial scratching would theoretically not be indicated or potentially deleterious. If the endometrial scratching was recommended and performed, on the subsequent embryo transfer cycle the clinical pregnancy rate was 47% (181/384) and the ongoing pregnancy rate at 12 weeks was of 38.5% (148/384). As a reminder, these patients failed to implant previously despite repeated attempts, representing an average of 8.3 good quality embryos transferred. In our experience, no complication - septic, haemorrhagic or perforation - was reported after the diagnostic biopsies or endometrial scratchings, which were performed with a pipelle (31, 32).

## Conclusion

Timed immune changes in the human endometrium are crucial for embryo implantation. These subtle cyclic modifications have been and keep being explored, with hormones, cytokines, immune cell populations and the emergence of potential new candidates including non-coding RNAs and micro-vesicles. But their usage as stable reproducible endometrial biomarkers is not widespread.

For the sake of clarity, we need to underline that the endometrial immune exploration during mid-luteal phase aims to confirm the switch of the immune cell populations or the balance of the cytokinic profile. These explorations differ from diagnostic tools used for endometritis (33), dysregulation of the local microbiome (34, 35) or the chase of the optimum synchrony for the timing of embryo implantation (36).

Endometrial immune profiling at the time of implantation window could help in selecting sub-groups of infertile patients who would benefit from a targeted intervention. In our experience, within a large population of patients having a history of unexplained repeated embryo implantation failure, only one third showed a low endometrial immune activation, potentially justifying an endometrial scratching.

We discussed the example of the diagnosed lack of local immune activation which could be supplied by an endometrial scratching. But our reflection should be wider on the need to determine the diagnosis of what we globally label as “embryo implantation failure”. Endometrial assessment should be part of the exploration of the failed initial foetal maternal dialogue and provide a rationale to the usage of potential targeted interventions. This vision can also promote the design of targeted studies of our therapeutic arsenal on selected cases.

With the development of embryo selection tools and the broader usage of donor gametes, unexplained repeated embryo implantation failure represents only a limited fraction of the patients in reproductive medicine, and the endometrial origin of this limitation can be proven in only a more limited number of cases. Now it is up to us to consider how we decide to face our failures and the energy we agree to spend on their exploration and correction.

## Author Contributions

All authors contributed to the article and approved the submitted version.

## Conflict of Interest

NL initiated the MatriceLAB Innove company and holds a patent covering the endometrial immune tests and the appended recommendations (PCT/EP2013/065355).

The remaining author declares that the research was conducted in the absence of any commercial or financial relationships that could be construed as a potential conflict of interest.
